# Dr. Lafuente's Method of Curing Yellow Fever

**Published:** 1805-12

**Authors:** 


### To the Editors of the Medical and Physical Journal.

GENTLEMEN,

THE medical world has already been informed, through the medium of several periodical publications, that Dr. Tadeo Lafuente, Physician to the Spanish Forces, and Inspector of the Public Health in the Camp before Gibraltar, intends shortly to publish a Treatise on a new Mode of preventing or curing the Yellow Fever. If you deem the following analysis of that Treatise, which has been printed by order of the Spanish Government, worthy of insertion in the Medical and Physical Journal, it is very much at your service.

Dr. Lafuente's great specific is bark, administered in large doses. The Doctor is aware that the public opinion, both at home and abroad, is rather unfavourable to his favorite medicine; and he is even inclined to admit, that there is some ground for the complaints daily preferred against the use of it; but then the fault lies not in the medicine itself, but in the mode of administering it. If the Doctor's own method be adopted, the cure of the yellow fever is equally certain with that of a tertian ague; and this method consists in giving the patient at least six or eight ounces of bark in the first forty-eight hours of the disease. Indeed, it is absolutely necessary to take the bark as soon as the symptoms of the fever make their appearance, without losing one moment, as appears from the following observations made in the village of Los Barrios, but little distant from St. Roque. There, says the Doctor, out of ninety patients, who took it between the first and the eighth hour of their falling sick, not one died, except a man who was carried off by the gout, under which he laboured at the same time. Of eight patients, to whom it was administered between the eighth and the tenth hour, all recovered. Of five, who began between the twelfth and the twenty-fourth hour, three recovered and two died. Of twenty, who never resorted to it till the second day, thirteen recovered, and seven died. Of seventeen, who waited till the third or fourth day, eight recovered and nine died. And, finally, out of eighty-nine who made no use of the bark, but took other medicines (sudorifics included) only twenty-two recovered, and the remaining seventy-seven died. Upon which the Doctor observes, that they who took the above quantity of the bark in the first ten hours, at once put a complete stop to the disease, and found themselves perfectly well and able to go out on the fourth day, without entering upon the second and most dangerous stage of the fever, which begins on the third day; whereas those who lost time, and entered more or less upon the second period, experienced its symptoms with more or less violence, and ran more or less danger, though they might recover in the end.

Dr. Lafuente and Don Joaquin de Bobadilla, Physician of Los Barrios, used, at first, to clear the stomach, by giving the patient some slight emetics; but the women and others, who being now acquainted with the efficacy of bark, often attempted to cure themselves without medical assistance, taught the Doctors not to lose one minute in these or other customary ceremonies, as they may be called. It was observed that the patients recovered, whether they took emetics or not, and that it was highly important to be cautious how they were used, as they took up a good deal of time, and left the stomach in such a state of irritation, that it could not keep the bark; in such a manner, says the Doctor, that, every doubt being now removed from the minds of the judicious part of the inhabitants, they contended, as it were, which should take most bark in the first forty-eight hours, or should begin earliest. No other medicine, therefore, was resorted to by the greatest number of patients, than getting half a pound or more of bark in powder, from the very moment they felt the first shiverings, and swallowing it in large spoons full, which might contain an ounce, or half an ounce, or at least three drachms of bark, every two hours, without losing time, or allowing themselves rest or sleep, either by night or day. Nay, there were some sick, who took ten, twenty, or even thirty-eight ounces of it in a few days, after a relapse brought on by carelessness or excesses, and sometimes without necessity, and merely for greater security. However, the Doctor advises such as wish to make a moderate use of the bark, to divide every ounce into three doses, and take one every two hours, and a little broth in the intervals; by which means they will take the said eight ounces in twenty-four doses, in the first forty-eight hours.

Should the patient throw up one of the doses, he ought to take another immediately, so that there may be no diminution of the abovementioned quantity of eight ounces, without allowing him to lose more than a quarter or half a quarter of an hour, to quiet the irritability of the stomach.

mach. If there had been any neglect, or time lost, he should take one dose of the bark, to be repeated in an hour afterwards, and at the third hour some broth may be given; going on in this manner, without allowing him one moment's rest, till the lost time be recovered, or the danger averted, which might have arisen from neglect or delay.

The vomiting which accompanies this disease, does not usually begin before the third day; and this is an additional reason why not a moment's time should be lost in the first forty-eight hours. To patients who, from a natural dislike to the bark, cannot retain it in powder, it is recommended to be administered in the form of pills. If, notwithstanding, it should still continue to be rejected, Dr. Lafuente recommends one or two table spoons full of the following mixture to be taken between the doses.

℞. Syrup. papaver albi ℥j. Spirit. cinnamomi ʒj. Vini, vel aquæ (ad libit. patient.) ℥vj. M. fiat mistura.

But Dr. Lafuente does not confine himself to laying his observations before the public, and proving their authenticity by a legal instrument, signed by twelve properly qualified individuals, he also confutes the objections which might be made against this method; and, among other assertions, maintains, that people of the most delicate constitutions may swallow as much bark as they chuse; that the fever, in its first period, is not inflammatory; and that though the copious and hasty administering of bark may bring on a temporary retention of urine, it may easily be cured by rubbing the lower part of the belly with oil, and ought not to be confounded with the suppression of urine, which is usually a mortal symptom in the second period of the disease.

The Doctor likewise mentions, that he, as well as the above named physician of Los Barrois, and a friar, chaplain to the infected, kept themselves free from the contagion, at the time that it raged with the greatest violence, and when they were constantly among the sick, by taking half an ounce of bark every morning; and that having at last agreed to discontinue their daily medicine, in the confidence that the disease could no longer prove fatal to them, they were taken ill a fortnight or three weeks after, but soon got rid of the fever by taking some bark in the first forty-eight hours.

And, finally, Dr. Lafuente maintains, that by sending the sick to well ventilated cottages in the country, instead of confining them in hospitals, or other regular buildings, not only the patients themselves have more chance of recovery, but the nurses who attend them need not be under any apprehensions of catching the fever, as there was no instance of any of them having been taken ill among no less a number than one hundred, who were employed in a lazaretto of cottages, eight or ten yards distant from each other, which was established in the neighbourhood of Los Barrios.

From the analysis here given, it must appear that the practice recommended by Dr. Lafuente is not altogether so new as he seems inclined to suppose, for it is well known, that British practitioners, both at home and abroad, have long been in the habit of prescribing the cinchona, in various malignant fevers, from the very commencement of the disease, and in as large doses as the stomach can bear, without any regard whatever to the state of pyrexia, particularly if, from the known character of the epidemic, considerable remissions are not soon to be expected, and that great danger is apprehended from repeated exacerbations. Throughout the whole plan of his treatment, he appears to have placed his sole reliance on the bark; and in his exhibition of it, to have ad hered with strict conformity to the practical rule of De Haen, "Neque pondus hic quicquam, aut mensura determinat, sed morbi levamen." Without entering at present into any particular examination of the author's mode of treatment, we shall only briefly observe, that while inattention to this rule has unquestionably proved the cause of its failure in cases wherein, otherwise, it might have succeeded, and that he acted judiciously in regulating the dose of the remedy in question, not by its absolute quantity, but by its relative effects, we cannot help expressing our surprise, that he never seems to have availed himself of the application of cold water, such a powerful and efficient remedy in abstracting redundant heat, and in dissevering the associated train of morbid febrile actions in the system.

R. H.

*Clement's Inn, October 20, 1805.*

